# Intravitreal Anti-VEGF Drugs and Signals of Dementia and Parkinson-Like Events: Analysis of the VigiBase Database of Spontaneous Reports

**DOI:** 10.3389/fphar.2020.00315

**Published:** 2020-03-12

**Authors:** Janet Sultana, Giulia Scondotto, Paola Maria Cutroneo, Francesca Morgante, Gianluca Trifirò

**Affiliations:** ^1^ Department of Biomedical and Dental Sciences and Morpho-functional Imaging, University of Messina, Messina, Italy; ^2^ Unit of Clinical Pharmacology, A.O.U. “G. Martino”, Messina, Italy; ^3^ Sicilian Regional Pharmacovigilance Centre, Messina, Italy; ^4^ Department of Clinical and Experimental Medicine, University of Messina, Messina, Italy; ^5^ Neurosciences Research Centre, Molecular and Clinical Sciences Research Institute, St George’s University of London, London, United Kingdom; ^6^ Department of Medical Informatics, Erasmus Medical Centre, Rotterdam, Netherlands

**Keywords:** pharmacovigilance, VigiBase, Alzheimer’s disease, Parkinson’ disease, dementia, neurodegeneration, antivascular endothelial growth factor drugs, intravitreal

## Abstract

**Introduction:**

Since vascular endothelial growth factor (VEGF) regulates several aspects of the central nervous system, particularly in dopaminergic neurons, VEGF inhibitors may be linked to Parkinson-like events and dementia, or variants of these diseases. Two recent case reports have found a potential link between intravitreal anti-VEGF use and Parkinson’s disease (PD) and dementia.

**Aim:**

To evaluate disproportionality in a large spontaneous reporting database concerning intravitreal anti-VEGF drugs and PD or dementia, and related conditions.

**Methods:**

Using VigiBase, individual case safety reports (ICSRs) attributed to intravitreal ranibizumab, aflibercept, pegaptanib, and bevacizumab were identified from 2010 to 2016. Within Standardised Narrow Medical Dictionary for Regulatory Activities (MedDRA^®^) Queries (SMQs) for “Parkinson-like events” and “Dementia,” suspected events were identified using preferred terms (PTs). The Proportional Reporting Ratio (PRR) was estimated with the lower 95% confidence intervals (CIs) for all drug-event pairs with ≥3 suspected events. The vigiGrade completeness score was reported for the ICSRs. The analyses were repeated, including only persons aged 65 and over.

**Results:**

Out of 18.9 million ICSRs, 7,945 (0.004%) concerned intravitreal anti-VEGF drugs. Of these, 27 (0.34%) were identified concerning the SMQs “Dementia” (N = 17, 62.96%) and “Parkinson-like events” (N = 10, 37.94%) in persons of all ages. Among persons age 65 and over, 4,758 (59.88% of relevant ICSRs) ICSRs were identified for anti-VEGF drugs. When restricting disproportionality analysis to persons aged 65 and over, no disproportionality was seen for any of the drug-event pairs at the level of SMQ. However, on analysing disproportionality by PT, a potential signal emerged for intravitreal ranibizumab and Parkinson’s disease [N = 6 ICSRs; PRR: 3.05 (95% CI: 1.36-6.81)]. In general, the vigiGrade completeness score was low for all the ICSRs of interest, as no ICSR had a score >0.8.

**Conclusion:**

Present findings suggest a potential signal for Parkinson’s disease related to intravitreal ranibizumab. This is supported by several biologically plausible mechanisms but requires confirmation through pharmacoepidemiological studies, especially because of the low number of cases.

## Highlights

- Although the ophthalmological safety profile of intravitreal anti-VEGF drugs has been described in detail, their systemic safety, including the risk of neurodegenerative diseases, is still coming to light.
- Among 7,945 case reports, of which 4,758 (59.9%) concerned persons aged 65 and over, for intravitreal ranibizumab, aflibercept, pegaptanib, or bevacizumab in VigiBase from 2010–2016, we identified nine potential cases of dementia and eight potential cases of Parkinson-like events.
- A potential signal concerning Parkinson’s disease was identified in relation to intravitreal ranibizumab use.

## Introduction

Vascular endothelial growth factor (VEGF) plays an important role in regulating physiological angiogenesis (Ferrara et al., 2003), inducing vascular permeability and promoting the survival of newly formed blood vessels (McColl et al., 2004). To date, six different VEGF isoforms have been identified (Robinson and Stringer, 2001). Retinal vascular diseases, such as age-related macular degeneration (AMD), diabetic macular oedema (DME), retinal vein occlusion (RVO), and choroidal neovascularization in myopic patients (mCNV), are the most common causes of vision loss in developed countries (Wong et al., 2014). Retinal hypoxia is the driver of these diseases, leading to elevated levels of hypoxia-inducible factor-1 (HIF-1), which in turn stimulates over-expression of VEGF (Campochiaro, 2013). The inhibition of VEGF is therefore a key pharmacological objective in these diseases (Ferrara, 2002).

Three anti-VEGF drugs, ranibizumab, aflibercept and pegaptanib, are currently marketed in Europe and North America for the intravitreal treatment of the above-mentioned retinal diseases, while bevacizumab is commonly used off-label for retinal vascular diseases, as its main indication is oncological. Although these anti-VEGF drugs are injected locally, they can lead to a large reduction in systemic plasma free-VEGF levels; a prospective clinical study found that a subset of patients were found to have a significant reduction in VEGF levels over three monthly intravitreal anti-VEGF administrations, persisting up to one month after treatment (Avery et al., 2017). The marked systemic suppression of VEGF may have implications for the systemic safety of anti-VEGF drugs. Indeed, a meta-analysis of randomized clinical trials (RCTs) on systemic adverse events showed that intravitreal anti-VEGF drugs, specifically ranibizumab, were associated with an increased risk of non-ocular haemorrhage (Thulliez et al., 2018). Findings by Thulliez et al. were in line with findings of a previous meta-analysis of RCTs, suggesting that long-term intravitreal ranibizumab users were at risk of systemic vascular adverse events (Ueta et al., 2014). Both meta-analyses suggested that the systemic ADRs investigated in clinical trials were more likely to occur in patients with AMD. The many limitations of RCTs in capturing ADRs (Sultana et al., 2013) highlight the importance of confirming such safety issues using real-world studies. An Italian nationwide pharmacovigilance study confirmed the plausibility of intravitreal anti-VEGF drugs being associated with systemic ADRs, including arterial thromboembolic events, such as stroke and myocardial infarction (Cutroneo et al., 2017). In addition to these ADRs, it has recently come to light that anti-VEGF drugs could be a risk factor for neurodegenerative processes.

Two recent case reports concerning intravitreal anti-VEGF use, one from Italy (Trifirò et al., 2018) and one from Germany (Meyer et al., 2018), identified a potential link with Parkinson’s disease and cognitive impairment, respectively. The former case report suggests a link between repeated intravitreal use of ranibizumab and Parkinsonism in an elderly patient with wet AMD (Trifirò et al., 2018). The other case report describes anterograde amnesia and confusion occurring with repeated intravitreal administration of ranibizumab followed by bevacizumab (Meyer et al., 2018). Indeed, an *in vitro* study examined the effect of VEGF on cellular processes or factors that modulate neuron survival (e.g., expression of proteins favoring or attenuating apoptotic processes and release of neurotrophic or neurotoxic factors), as well as on neuron survival directly. This study found that VEGF improved neuron survival in a dose-dependent manner; this correlated with a reduction in apoptosis-inducing proteins, an increase in apoptosis-preventing proteins and neurotrophic factors such as pigment epithelial-derived factor and a reduction in amyloid beta, which is the neurotoxic protein involved in Alzheimer’s disease (Sanchez et al., 2010).

Except for these case studies and one *in vitro* experimental study, the evidence on the potential link between anti-VEGF drugs and neurodegeneration is poor. The aim of the present study was therefore to investigate the potential association between the use of intravitreal anti-VEGF drugs and dementia and Parkinson-related disorders using VigiBase, the World Health Organization (WHO) international spontaneous reporting database, by calculating Proportional Reporting Ratios as a disproportionality measure.

## Methods

### Data Source

VigiBase, the WHO global database of individual case safety reports (ICSRs), was used as a data source. VigiBase is the largest spontaneous ADR report database worldwide, founded in 1968 by the WHO Programme for International Drug Monitoring, managed by the Uppsala Monitoring Centre (UMC). VigiBase contains anonymised information on suspected ADR reports associating one or more medicinal products to one or more suspected ADRs. It currently contains over 20 million reports collected globally. This database has been described in detail elsewhere (Lindquist, 2008). Data for the present study was obtained from 2010–2016.

### Study Drugs

For the present study, only ICSRs attributed to intravitreal anti-VEGF drugs, whether with licensed ophthalmologic indications or off-label, were identified and selected. During the study period, the following drugs were available on market and were therefore included in the study: aflibercept, ranibizumab, bevacizumab, and pegaptanib. In addition, the intravitreal implant of the corticosteroid dexamethasone was also considered as a separate exposure of interest, as this has a major overlapping indication of use (DME) with intravitreal anti-VEGF drugs and a similar route of administration. ICSRs where route of drug administration was not specifically reported were excluded. All study drugs were identified by generic name.

### Suspected Events

**Figure 1:**
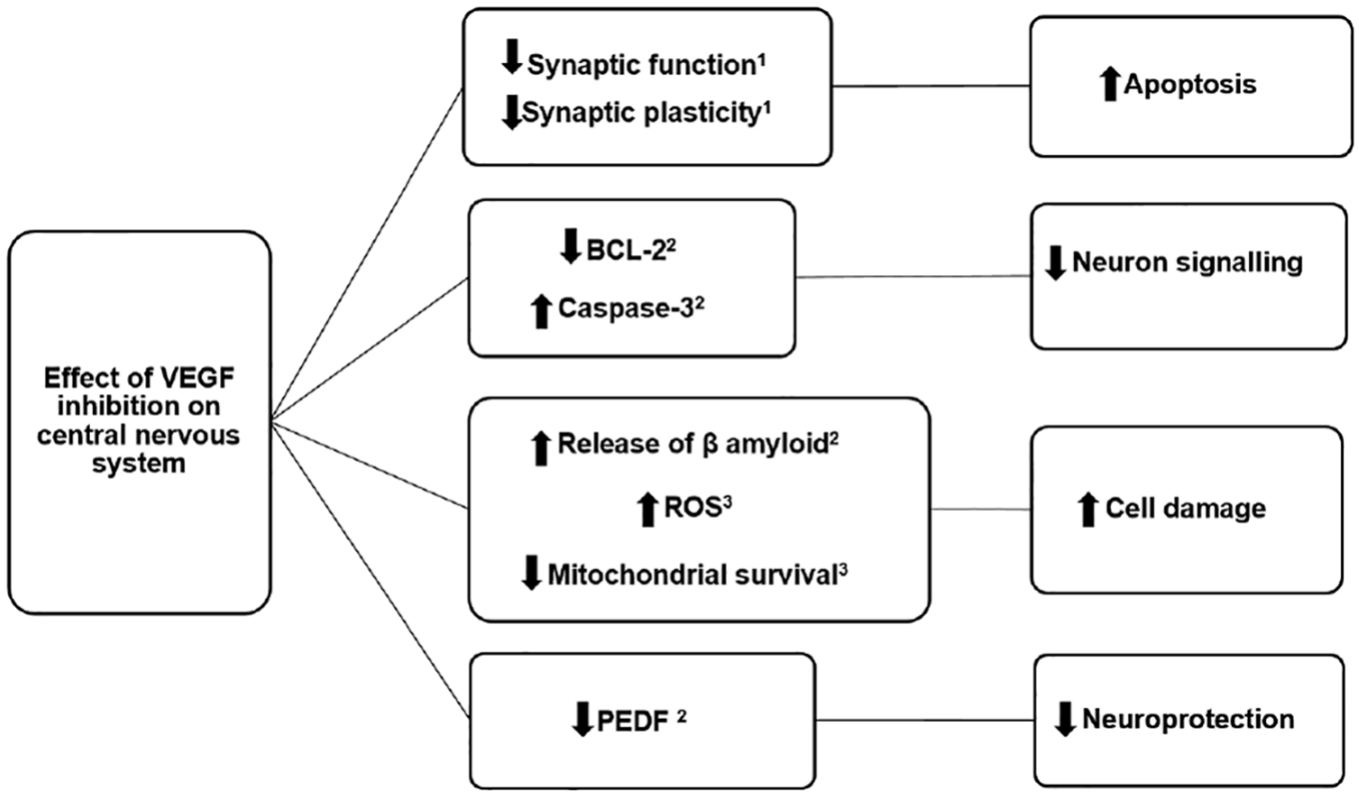
Possible mechanisms of action by which intravitreal antivascular endothelial growth factor drugs could increase the risk of neurodegenerative disease such as Parkinson’s disease and Alzheimer’s disease. ROS, reactive oxygen species; PEDF, pigment epithelium-derived factor; VEGF, vascular endothelial growth factor. (Sanchez et al., 2010; De Rossi et al., 2016; Caballero et al., 2017).

The present study focused on two neurodegenerative diseases and related symptoms, i.e., Parkinson-like events and dementia. Suspected events were first identified using Standardised Narrow Medical Dictionary for Regulatory Activities (MedDRA^®^) Queries (SMQs) for “Parkinson-like events” and “Dementia” (**Online Resource**, **Table 1**). SMQs are composed of preferred terms (PTs) that refer to a range of clinical concepts, including syndromes, well-defined diseases and symptoms. A single ICSR may have more than one SMQ and/or more than one PT per each SMQ. As a result, the number of PTs does not always correspond to the same number of SMQs. The diversity of concepts between SMQs and PTs gave rise to potential conceptual overlap which is inherent to the MedDRA^®^ classification system. In addition, PTs that fall under an SMQ do not all necessarily have the same level of clinical detail, as some refer to diseases while others to symptoms. A hierarchical concept map was created to provide an overview of the level of conceptual clinical detail encompassed by all the SMQs and PTs considered in the present study (**Online Resource**, **Figure 1**). We included only suspected events related to anti-VEGF drugs administered intravitreally.

**Table 1:** Individual case safety reports concerning intravitreal antivascular endothelial growth factor or dexamethasone implant use, grouped by standardized MedDRA^®^ query, in patients of all ages.

Intravitreal drug	SMQ	N	PRR (95% CI)
Aflibercept	Dementia	**6**	**2.78 (1.25–6.21)**
	Parkinson-like events	3	0.65 (0.21–2.02)
Ranibizumab	Dementia	**11**	**2.50 (1.38–4.52)**
	Parkinson-like events	6	0.64 (0.28–1.42)
Pegaptanib	Dementia	–	–
	Parkinson-like events	–	–
Bevacizumab	Dementia	–	–
	Parkinson-like events	1	NA
Dexamethasone	Dementia	–	–
	Parkinson-like events	–	–

Signals of disproportionate reporting are highlighted in bold where statistical significance is reached and more than three cases were identified. N, number; NA, not assessable; CI, confidence interval; PRR, Proportional Reporting Ratio; SMQ, standardized MedDRA^®^ queries.

### Data Analysis

#### Data Screening Method

To detect potential safety signals, the main method of disproportionality analysis used was the Proportional Reporting Ratio (PRR), along with lower 95% confidence intervals (95% CI). PRR is a widely used measure of association applied to spontaneous reports databases (Evans et al., 2001). The PRR was calculated using as comparison group all other reports for any drugs administered by any route in VigiBase from 2010–2016, first in persons of all ages then restricting only to persons aged 65 and over. This restriction was carried out in order to reduce confounding due to age because dementia and Parkinson-like events are more frequent in elderly persons compared to younger ones. For a signal to emerge in this population of elderly persons, the events of interest have to be more common than the background reporting of these events among elderly persons. PRR was only calculated where three or more events of interest were identified. Where the PRR was significant, the following information was extracted from the original ICSRs, where available: sex, age at reporting date, indication of drug use, time to suspected event onset (i.e., time elapsed between the date of drug initiation and the date of event reported) and causality assessment. Information on dose was not collected, as there as is only one strength available for each drug. Furthermore, only where the PRR was significant, two other measures of disproportionality were calculated to confirm the results: the reporting odds ratio (ROR) and information component (IC). ROR was presented with 95% CI while IC was presented with IC_025,_ i.e., the lower end of the 95% credibility interval. All analyses were carried out clustering ICSRs first by SMQ and separately, by PT.

The causality assessment was carried out for each report where the PRR was significant, using the WHO-UMC system for standardised case causality assessment, resulting in the following likelihood of causality being assigned: certain, probable/likely, possible, unlikely, conditional/unclassified, and unassessable/unclassifiable. The vigiGrade completeness score, developed by the UMC, was used to measure the completeness of the ICSRs. This score takes into account the completeness of the following fields: time-to-onset, age, sex, indication, outcome, report type, dose, country, primary reporter, and comments. The best score possible is 1 but scores >0.8 are nevertheless considered to denote a good level of completeness (Bergvall et al., 2014).

Further analyses were conducted where the PRR was significant, such as stratification of results by age (65–74 years and ≥75 years) and by sex.

### Ethics Approval and Data Protection

The present study did not require ethics approval as it was conducted using retrospectively collected, de-identified data. Descriptive patient-level tables containing information (e.g., sex, age, country of origin, and indication of drug use) on 10 patients or less were not presented to further protect patient privacy.

## Result

### Signal Detection

During the study period, there were over 18.9 million ICSRs in VigiBase of which 7,945 (0.004%) ICSRs concerned intravitreal anti-VEGF drugs and any suspected event among persons of all ages. From this pool of ICSRs for intravitreal anti-VEGF drugs, 27 (0.33%) were identified concerning the narrow SMQs for “Parkinson-like events” and “Dementia” which were potentially related to intravitreal anti-VEGF drugs. No reports were found concerning dexamethasone intravitreal implant and the events in question. Concerning intravitreal anti-VEGF drugs, 10 ICSRs for the SMQ “Parkinson-like events” and 17 ICSRs for the SMQ “Dementia” were identified for persons of all ages. The disproportionality analysis carried out at level of the SMQs suggested a statistically significant PRR for intravitreal ranibizumab use in relation to “Dementia” 2.50 (95% CI: 1.38–4.52), with N = 11 ICSRs. Similarly, a significant PRR was observed for aflibercept in relation to the SMQ “Dementia” [PRR: 2.78 (95% CI: 1.25–6.21)], with N = 6 ICSRs. No statistically significant signal of disproportionality was found for any of the anti-VEGF drugs included in the study and the SMQ “Parkinson-like events” (**Table 1**). On increasing the specificity of the suspected events by conducting analyses at the level of PT, a trend of disproportionality among persons of all ages emerged for one intravitreal anti-VEGF drug and “Parkinson’s disease”, specifically ranibizumab, with a PRR of 3.11 (95% CI: 1.39–6.94) concerning N=6 ICSRs. The disproportionality analysis among PTs making up the narrow SMQ “Dementia” yielded a potential signal for ranibizumab and the PT “Dementia, Alzheimer type” [N = 5, with a PRR of 4.55 (95% CI: 1.89–11.0)] (**Table 2**). Disproportionality was also identified for intravitreal aflibercept and the PT “Dementia” [N = 5, PRR: 3.37 (95% CI: 1.40–8.14)].

**Table 2:** Individual case safety reports for intravitreal ranibizumab, aflibercept, and bevacizumab grouped by preferred term related to dementia and/or Parkinson-like events standardized MedDRA queries, in patients of all ages.

SMQ	Intravitreal drug	PT	N	PRR (95% CI)
Dementia	Aflibercept	Dementia	**5**	**3.37 (1.40–8.14)**
		Senile dementia	1	NA
	Ranibizumab	Dementia, Alzheimer’s type	**5**	**4.55 (1.89–11.0)**
		Mixed Dementia	1	NA
		Dementia	6	1.98 (0.89–4.42)
Parkinson-like events	Aflibercept	Akinesia	1	NA
		Parkinson’s disease	2	NA
	Ranibizumab	Hypertonia	2	NA
		Parkinson’s disease	**6**	**3.11 (1.39–6.94)**

Signals of disproportionate reporting are highlighted in bold where statistical significance is reached and more than three cases were identified. CI, confidence interval; N, number; NA, not assessable; PT, preferred term; PRR, Proportional Reporting Ratio; SMQ, standardized MedDRA^®^ queries.

Among elderly persons, a total of 4,758 (59.9% of all anti-VEGF related reports) ICSRs were identified, eight (0.2%) concerning the SMQ “Dementia” and nine (0.2%) ICSRs for the SMQ “Parkinson-like events.” When restricting disproportionality analysis to persons aged 65 and over, no disproportionality was seen for any of the drug-event pairs at the level of SMQ (**Table 3**). However, on analysing disproportionality by PT, a potential signal emerged for ranibizumab and Parkinson’s disease [N = 6 ICSRs; PRR: 3.05 (95% CI: 1.36–6.81)] (**Table 4**).

**Table 3:** Individual case safety reports for intravitreal ranibizumab and aflibercept in persons aged 65 and over, grouped by standardized MedDRA^®^ query related to dementia and/or Parkinson-like events standardized MedDRA queries.

**SMQ**	**Intravitreal drug**	**N**	**PRR (95% CI)**
Dementia	Aflibercept	1	–
	Ranibizumab	8	1.36 (0.68–2.72)
Parkinson-like events	Aflibercept	2	NA
	Ranibizumab	6	0.85 (0.38–1.89)

CI, confidence interval; N, number; PRR, Proportional Reporting Ratio; SMQ, standardized MedDRA^®^ queries.

**Table 4:** Individual case safety reports for intravitreal ranibizumab grouped by preferred term, in patients aged 65 years or older.

PT	Drug	N	PRR (95% CI)
Parkinson’s disease	Ranibizumab	**6**	**3.05 (1.36–6.81)**
	Aflibercept	1	NA
Hypertonia	Ranibizumab	2	NA
Akinesia	Aflibercept	1	NA
Dementia	Ranibizumab	5	1.24 (0.51–3.00)
	Aflibercept	1	NA
Mixed dementia	Ranibizumab	**1**	NA

Signals of disproportionate reporting are highlighted in bold where statistical significance is reached and more than three cases were identified. CI, confidence interval; N, number; NA, not assessable; PT, preferred term; PRR, Proportional Reporting Ratio.

A non-significant trend of disproportionality concerning dementia was seen both at the level of SMQ [1.36 (95% CI: 0.68-2.72)] and at the level of PT [1.24 (95% CI: 0.51–3.00)] in relation to intravitreal ranibizumab use. When the ICSRs for the ranibizumab-Parkinson’s disease PT were stratified by age group (65–74 and >74) and sex, a trend of disproportionality was seen for PRR and ROR while the IC suggested a very weak, borderline disproportionality for persons aged 65 to 74. It was very difficult to interpret these results reliably due to the small number of ICSRs (**Online Resource**, **Table 2**).

### Case Description for Safety Signals

The patient-level case description for the six ICSRs was not presented in tabular format to protect patients’ privacy. Within the SMQ “Parkinson-like events”, six ICSRs were identified for the PT “Parkinson’s disease” in relation to intravitreal ranibizumab. The mean age for patients developing “Parkinson’s disease” after exposure to ranibizumab was 76.5 ± 5.6; age was not missing any of these ICSRs. Overall, for the PT “Parkinson’s disease,” 83% of persons exposed to ranibizumab in the ICSRs were males; no missing gender information was identified for this drug-event pair. The mean time to onset was estimable for four out of six ICSRs, and was 2.3 ± 2.0 years. For the six ICSRs containing the PT “Parkinson’s disease,” the causality assessment for ranibizumab was “possible” for five (83.33%) ICSRs and “unlikely” for one (16.66%) ICSR. Causality was always assessable for this PT. In general, the vigiGrade completeness score was low for all the ICSRs of interest, as not a single ICSR had a score >0.8. The median (1^st^–3^rd^ quartile) value was 0.58 (0.30–0.71).

## Discussion

The main finding from this study was the identification of a potential signal related to the occurrence of Parkinson’s disease with intravitreal ranibizumab use. The present study is the first to evaluate the occurrence of this signal. The reason why disproportionality was significant only concerning ranibizumab is very unlikely to be explained exclusively by its mechanism of action, which is almost identical to other anti-VEGF drugs (Avery et al., 2017). A more likely explanation is the low statistical power achieved in the study. Furthermore, intravitreal ranibizumab has been on the market longer than other drugs, such as aflibercept, and it is more commonly used than pegaptanib, which has fewer ophthalmologic indications than ranibizumab. As a result, it is possible that the number of persons exposed to ranibizumab is much higher than other anti-VEGF drugs. The reason why disproportionality was not seen for dementia may be related to the high background reporting rate of this event in elderly persons as well as the low number of cases identified. Nevertheless, a trend suggesting non-significant disproportionality was identified for intravitreal ranibizumab and dementia among elderly persons.

The neurological symptoms of the potentially intravitreal anti-VEGF induced Parkinsonism outlined in a case report a few years ago were described in great detail, suggesting that this kind of Parkinsonism may present with atypical features, such lack of cortical and subcortical atrophy (Trifirò et al., 2018). The authors of this case report note that idiopathic PD cannot be excluded, since several neurologic features are consistent with this diagnosis, including vascular lesions in the basal ganglia and DAT SPECT results showing pre-synaptic dopamine loss. On the other hand, the case report concerning the occurrence of amnesia with intravitreal bevacizumab and ranibizumab use did not provide such a high level of neurological detail as the case report by Trifirò et al. and as a result, it is difficult to understand whether the pathophysiological context is consistent with amnesia occurring to due neurodegenerative processes (Meyer et al., 2018). Meyer et al. report that the patient complained of mild memory impairment prior to treatment with anti-VEGF drugs, with a cranial MRI of the patient suggesting moderate brain atrophy prior to intravitreal anti-VEGF use. However a mini-mental state exam (MMSE) indicated almost intact cognitive function (29 points out of 30). It is therefore unlikely that pre-existing cognitive impairment explains the acute confusional state which developed after intravitreal anti-VEGF use. Meyer et al. report that on discontinuing intravitreal anti-VEGF treatment, no further amnesia developed and that cranial MRI two years after the first intravitreal anti-VEGF exposure did not show any significant differences compared to before treatment, presumably in terms of atrophy (a detailed description of the results was not available). From this case report, the plausibility of anti-VEGF drugs as a risk factor for dementia in a neurodegenerative context, i.e. a progressive and long-term symptom, is not clear and requires further elucidation.

The plausibility of the association between intravitreal anti-VEGF drugs and neurodegenerative diseases such as Parkinson’s disease is supported by a large body of pre-clinical evidence. Firstly, there are several biologically plausible mechanisms by which the study drugs could have led to or contributed to neuropathological processes common to both Parkinson’s disease and and to other neurodegenerative diseases (**Figure 1**). The inhibition of VEGF is known to lead to a reduction in synaptic function and plasticity, leading to impaired neuron signalling (Sharma et al., 2019). Furthermore, inhibition of VEGF can lead to a reduction in Bcl-2 gene expression and increase in the protein caspase-3, with both these two effects leading to increased cell death (Sanchez et al., 2010). Antagonism of VEGF receptors has also been linked to an increase in free-radical oxygen species with resulting damage to mitochondria (De Rossi et al., 2016) as well as stimulating the release of β-amyloid, a protein which is the hallmark of Alzheimer’s disease and can co-occur in Parkinson’s disease (Sanchez et al., 2010; Lim et al., 2019). Although one of the dementia-related PTs in the signals identified did not specifically refer to Alzheimer’s disease, this particular type of dementia is the most frequent subtype, known to account for 60-80% of all dementia cases (Caballero et al., 2017). We therefore expect that the majority of identified non-specific dementia events will likely refer to Alzheimer’s disease.

Concerning the risk of Parkinson’s disease, it is known that VEGF administration inhibits loss of dopaminergic neurons in experimental models, particularly in the substantia nigra and in the striatum (Kumar and Tsao, 2018). A reduction in plasma free-VEGF levels also leads to a reduction in pigment epithelium-derived factor (PEDF), a neuroprotective protein that promotes cell survival (Sanchez et al., 2010). In addition, low VEGF levels may impair neural tissue perfusion, causing ischaemia and production of free radicals (Sheikh et al., 2017). VEGF is known to be neuroprotective, inhibiting apoptosis, stimulating neurogenesis and activating antioxidants (Storkebaum and Carmeliet, 2004; Yasuhara et al., 2004). Specifically, ranibizumab and aflibercept bind to VEGF-A, one of the five isoforms of the VEGF family and one of the strongest inducers of vascular permeability (Zachary, 2005). VEGF-A has been shown to have neuroprotective effects in several *in vitro* and *in vivo* PD models (Storkebaum and Carmeliet, 2004; Ylä-Herttuala et al., 2007).

In addition, neovascular retinal disease such as AMD and DME are the most common indications of use for intravitreal anti-VEGF drugs, and are characterised by retinal neurodegeneration (de Jong, 2006; Tian et al., 2007) Indeed, AMD and DME share common biological pathways with neurodegenerative processes, such as oxidative stress and inflammation. Persons with DME specifically are at a higher risk of neurodegeneration, since diabetes and the insulin resistance that precedes it, are known to be closely linked biologically to neurodegeneration (Simó and Hernández, 2014).

Other factors supporting the link between intravitreal ranibizumab and the suspected events identified is the time to onset. While it was not possible to calculate this for any dementia-related PTs for ranibizumab or aflibercept, for the ranibizumab-Parkinson’s disease pair, the mean time to event, which was estimable for four out of six ICSRs, was 2.3 ± 2.0 years. We expect patients who developed these events to be treated chronically, since the indication of use in all cases where time to onset could be calculated was AMD, which generally requires long-term treatment. During this period, assuming treatment was in line with the recommendations of summary of product characteristics, the number of injections which a patient would have received two years after initiating intravitreal ranibizumab treatment would range from 8 to over 10, depending on the therapeutic approach used after a loading phase, i.e., treat and extend or as needed (Steen et al., 2005). Long-term use of intravitreal anti-VEGF drugs may have led to a persistent and prolonged suppression of VEGF levels, which may have in turn triggered neurodegenerative events in a population with a high baseline risk of such events. The combined long-term onset of the suspected neurological events observed and the expected high cumulative dose to which is patient is likely exposed may be arguments to support the role of anti-VEGF drugs in increasing the risk of the two neurodegenerative diseases under study.

While all of these findings appear to support a potential increased risk of neurodegeneration with intravitreal anti-VEGF drug use, it is likely that this risk is relevant mostly to persons who have a high baseline risk of neurodegeneration (e.g., family history of Alzheimer’s disease, Parkinson’s disease, uncontrolled diabetes, etc.) and persons at risk of excessive VEGF inhibition with normal doses. The clinical benefits of intravitreal anti-VEGF drugs are significant and any potential risk of neurodegeneration, if confirmed in large pharmacoepidemiology studies, should be weighed against the expected benefits of treatment.

The present study has several strengths as well as limitations. Spontaneous report analysis depends heavily on the completeness of the data recorded. Although most data for the six ICSRs of interest was recorded, age, sex, treatment initiation date, causality assessment, indication of use, and dose were often not available for other ICSRs. Indeed, the vigiGrade completeness score was generally low. The ICSRs that make up VigiBase are sent to the UMC from a variety of sources, which may have varying quality of reporting. Furthermore, there may also be a variation in national pharmacovigilance methods to assess causality. To address these limitations, we evaluated causality using a single algorithm, i.e., WHO-UMC system for standardized case causality assessment, to avoid potentially differential misclassification. While we hypothesise that the suspected events observed may have been due to cumulative exposure to anti-VEGF drugs, we cannot be certain of this since the number of previously administered injections is not known. Finally, another limitation is that although we used the narrowest possible SMQ terms, the SMQs for “Dementia” and “Parkinson-like events” do not refer to specific, well-classified neurodegenerative diseases. Similarly, some of the PTs also do not refer to specific neurodegenerative diseases but rather to symptoms of these diseases. For signal concerning well-defined PTs (e.g., Alzheimer’s disease and Parkinson’s disease), we do not have information on whether the conditions are long-lasting and irreversible, criteria that are important to correctly identify neurodegenerative diseases. Findings from the present study must be interpreted in light of its limitations.

Despite the limitations of the data sources used and of the analyses conducted, the identification of a potential signal highlights the importance of conducting high-powered pharmacoepidemiological studies to investigate the association between neurodegenerative disorders and long-term intravitreal anti-VEGF use. The importance of such studies is that they can overcome several of the limitations inherent to analyses of spontaneous ADR databases, such as potential differential reporting for specific drugs and underreporting, which may be high given that neurodegenerative diseases are common among users of intravitreal anti-VEFG drugs, who are generally elderly. Another important limitation is the inability to adjust for confounders and especially, the lack of a denominator. In addition, pharmacoepidemiological studies conducted using claims databases and/or electronic medical records can also provide detailed information on exposure, including the temporal relationship between the exposure and the event of interest.

## Conclusion

Present findings suggest a potential signal for Parkinson’s disease related to intravitreal ranibizumab use. The plausibility of these signals is supported by the biological events downstream of VEGF inhibition, leading to a reduction in synaptic function and plasticity, impaired neuron signalling and increased apoptosis. However, the potential signal identified requires confirmation by pharmacoepidemiological studies. On the other hand, should this signal be confirmed, it is important to weigh anti-VEGF drug risks against their benefits, as these drugs are very effective in preventing or slowing down blindness among persons with retinal neovascular disease.

## Data Availability Statement

Restrictions apply to the datasets: The datasets for this article are not publicly available because they are not owned by the authors of this paper. Requests to access the datasets should be directed to the Uppsala Monitoring Centre at ADRinfo@who-umc.org.

## Author Contributions

GT conceived the study. JS, GS, and GT contributed to the study design. Data analysis was curated by GS. The first draft of the manuscript was written by JS and GS. PC, FM and GT critically revised the manuscript. All authors read and approved the final manuscript.

## Funding

This study received funding from the Italian Medicines Agency through the grant “Short- and long-term monitoring of the benefit-risk profile of intravitreal use of anti-VEGF drugs through network of clinical and administrative data” (CUP-H56D16000070005), awarded to Gianluca Trifirò (University of Messina).

## Conflict of Interest

GT attended in the last years advisory boards on topics not related to this presentation and organized by Sandoz, Hospira, Sanofi, Biogen, Ipsen, Shire and is consultant for Otsuka. He is principal investigator of observational studies funded by several pharmaceutical companies (e.g., Amgen, AstraZeneca, Daiichi Sankyo, IBSA) to University of Messina as well as scientific coordinator of the Master program “Pharmacovigilance, pharmacoepidemiology and pharmacoeconomics: real world data evaluations” at University of Messina which is partly funded by several pharmaceutical companies.

The remaining authors declare that the research was conducted in the absence of any commercial or financial relationships that could be construed as a potential conflict of interest.
