# Supplementary material for: Intravitreal Anti-VEGF Drugs and Signals of Dementia and Parkinson-Like Events: Analysis of the VigiBase Database of Spontaneous Reports
Source: Front Pharmacol. 2020 Mar 12;11:315. doi: 10.3389/fphar.2020.00315 (PMC7080978; doi:10.3389/fphar.2020.00315)
Supplement: Supplementary file 1 [file DataSheet_1.pdf]

Supplementary material

Table 1 Narrow Standardised MedDRA Queries with their respective Preferred Terms for dementia-related events and Parkinson-related events for the study outcomes of interest

	**Dementia: narrow SMQ**	**Parkinson-like events: narrow: SMQ**
**Preferred Term**	Clinical dementia rating scale score abnormal	Akinesia
	Corticobasal degeneration	Bradykinesia
	Creutzfeldt-Jakob disease	Cogwheel rigidity
	Dementia	Freezing phenomenon
	Dementia Alzheimer's type	Hypertonia
	Dementia of the Alzheimer's type, uncomplicated	Hypertonia neonatal
	Dementia of the Alzheimer's type, with delirium	Muscle rigidity
	Dementia of the Alzheimer's type, with delusions	On and off phenomenon
	Dementia of the Alzheimer's type, with depressed mood	Parkinsonian crisis
	Dementia with Lewy bodies	Parkinsonian gait
	Early onset familial Alzheimer's disease	Parkinsonian rest tremor
	Frontotemporal dementia	Parkinsonism
	Hippocampal sclerosis	Parkinsonism hyperpyrexia syndrome
	Korsakoff's syndrome	Parkinson's disease
	Mini mental status examination abnormal	Parkinson's disease psychosis
	Mixed dementia	Resting tremor
	Presenile dementia	
	Prion disease	
	Progressive supranuclear palsy	
	Scatolia	
	Senile dementia	
	Variant Creutzfeldt-Jakob disease	

Abbreviations: SMQ: Standardised MedDRA® Queries
