# Supplementary material for: Intravitreal Anti-VEGF Drugs and Signals of Dementia and Parkinson-Like Events: Analysis of the VigiBase Database of Spontaneous Reports
Source: Front Pharmacol. 2020 Mar 12;11:315. doi: 10.3389/fphar.2020.00315 (PMC7080978; doi:10.3389/fphar.2020.00315)
Supplement: Supplementary file 2 [file DataSheet_2.pdf]

Supplementary material

Figure 1 Hierarchical concept map of terms covered by SMQs and PTs included in the study

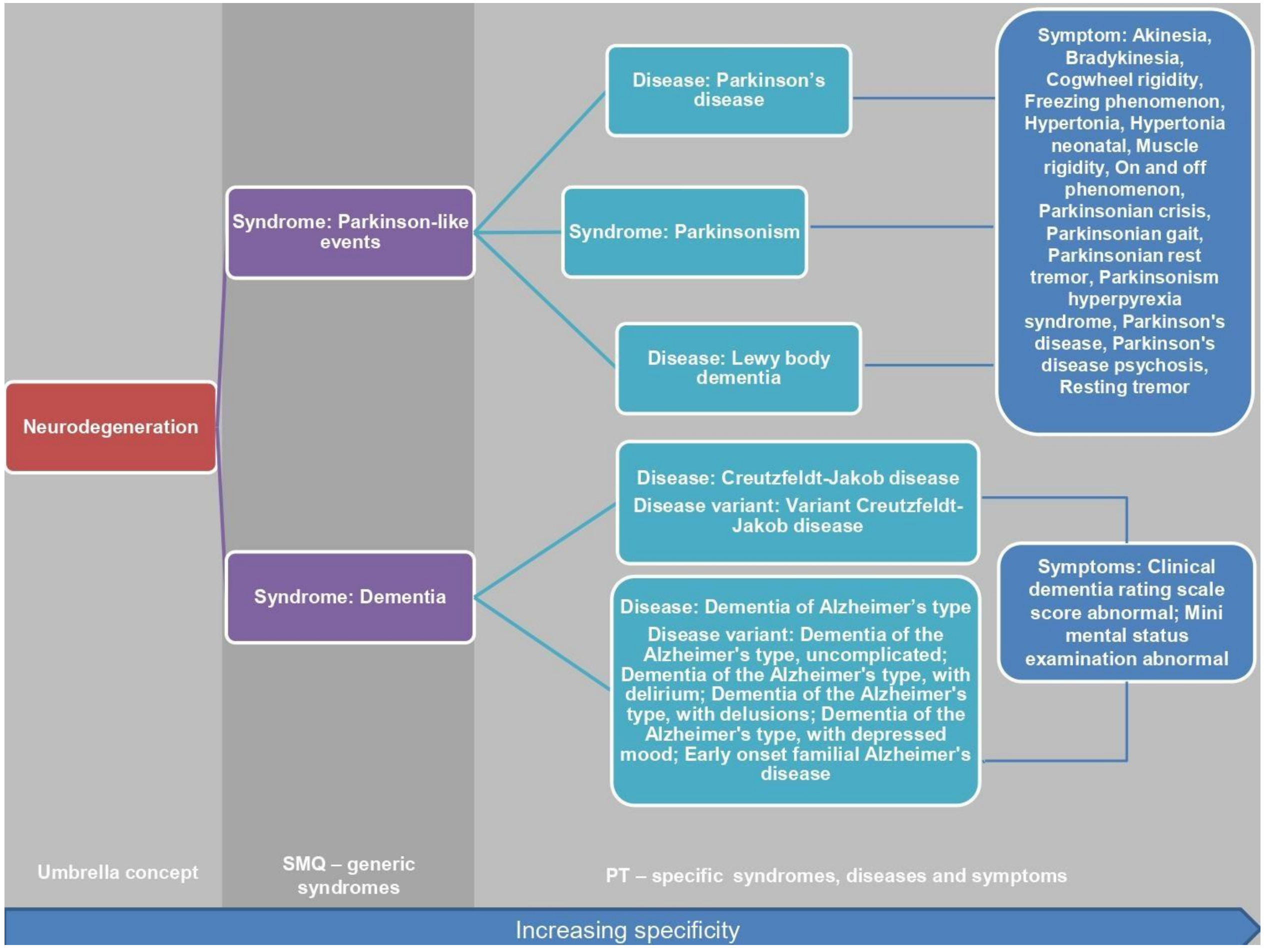

Abbreviations: SMQ: Standardised MedDRA® Queries; PT: Preferred Term
