# Supplementary material for: Intravitreal Anti-VEGF Drugs and Signals of Dementia and Parkinson-Like Events: Analysis of the VigiBase Database of Spontaneous Reports
Source: Front Pharmacol. 2020 Mar 12;11:315. doi: 10.3389/fphar.2020.00315 (PMC7080978; doi:10.3389/fphar.2020.00315)
Supplement: Supplementary file 3 [file DataSheet_3.pdf]

Supplementary Material

Table 2 Individual case safety reports for intravitreal ranibizumab-Parkinson's disease, stratified by age and sex

			**Drug-preferred term pair**			
			**Ranibizumab-Parkinson's disease**			
			**N**	**PRR (95% CI)**	**ROR (95% CI)**	**IC (IC_{25})**
**Unstratified**			6	**3.11 (1.40-6.94)**	**3.11 (1.40-6.94)**	1.42 (0.04)
**Stratified**	**Age**	65-74 years	4	**7.97 (2.99-21.25)**	**7.97 (2.99-21.25)**	2.16 (0.42)
		>75 years	2	1.24 (0.31-5.00)	1.24 (0.31-5.00)	0.24 (-2.34)
		Unknown	-	-	-	-
	**Sex**	Male	5	**4.38 (1.82-10.53)**	**4.38 (1.82-10.53)**	1.74 (0.21)
		Female	1	1.27 (0.18-9.05)	1.27 (0.18-9.05)	0.22 (-3.57)
		Unknown	-	-	-	-

Abbreviations: CI: confidence interval; IC: information component; PRR: Proportional Reporting Ratio; ROR: Reporting Odds Ratio
